# Pharmacokinetics and efficacy of 2-methoxyoestradiol and 2-methoxyoestradiol-*bis-*sulphamate *in vivo* in rodents

**DOI:** 10.1038/sj.bjc.6601591

**Published:** 2004-02-17

**Authors:** C R Ireson, S K Chander, A Purohit, S Perera, S P Newman, D Parish, M P Leese, A C Smith, B V L Potter, M J Reed

**Affiliations:** 1 Endocrinology and Metabolic Medicine and Sterix Ltd, Faculty of Medicine, Imperial College, St Mary's Hospital, London W2 1NY, UK; 2 Medicinal Chemistry and Sterix Ltd, Department of Pharmacy and Pharmacology, University of Bath, Claverton Down, Bath BA2 7AY, UK

**Keywords:** 2-methoxyoestradiol, 2-methoxyoestradiol-*bis-*sulphamate, breast cancer, sulphatase, sulphatase inhibitor, pharmacokinetics

## Abstract

2-Methoxyoestradiol (2-MeOE2) is an endogenous oestrogen metabolite that inhibits the proliferation of cancer cells *in vitro*, and it is also antiangiogenic. *In vivo* 2-MeOE2, when administered at relatively high doses, inhibits the growth of tumours derived from breast cancer cells, sarcomas and melanomas. Sulphamoylated derivatives of 2-MeOE2 are more potent inhibitors of *in vitro* breast cancer cell growth than 2-MeOE2. In the present study, we have compared the pharmacokinetic profiles and metabolism of 2-MeOE2 and its sulphamoylated derivative, 2-methoxyoestradiol-*bis*-sulphamate (2-MeOE2*bis*MATE), in adult female rats. Their ability to inhibit tumour growth was compared in nude mice bearing xenografts derived from MDA-MB-435 (oestrogen receptor negative) melanoma cancer cells. After a single oral 10 mg kg^−1^ dose of 2-MeOE2*bis*MATE, significant concentrations of this compound were still detectable at 24 h. In contrast, no 2-MeOE2 or metabolites were detected in plasma at any time after a 10 mg kg^−1^ oral dose. Thus, the bioavailability of 2-MeOE2 is very low, whereas for 2-MeOE2*bis*MATE it was 85%. No significant metabolites of 2-MeOE2*bis*MATE were detected in plasma after oral or intravenous dosing, showing that this drug is resistant to metabolism. In the tumour efficacy model, oral administration of 2-MeOE2*bis*MATE, at 20 mg kg^−1^ day^−1^ daily for 28 days, almost completely inhibited tumour growth. Inhibition of tumour growth was maintained for a further 28 days after the cessation of dosing. At this dose level, 2-MeOE2 did not inhibit tumour growth. The resistance to metabolism shown by 2-MeOE2*bis*MATE and its ability to inhibit tumour growth *in vivo* suggest that this compound should have considerable potential for development as a novel anticancer drug.

2-Methoxyoestradiol (2-MeOE2, Figure 1

**Figure 1:**
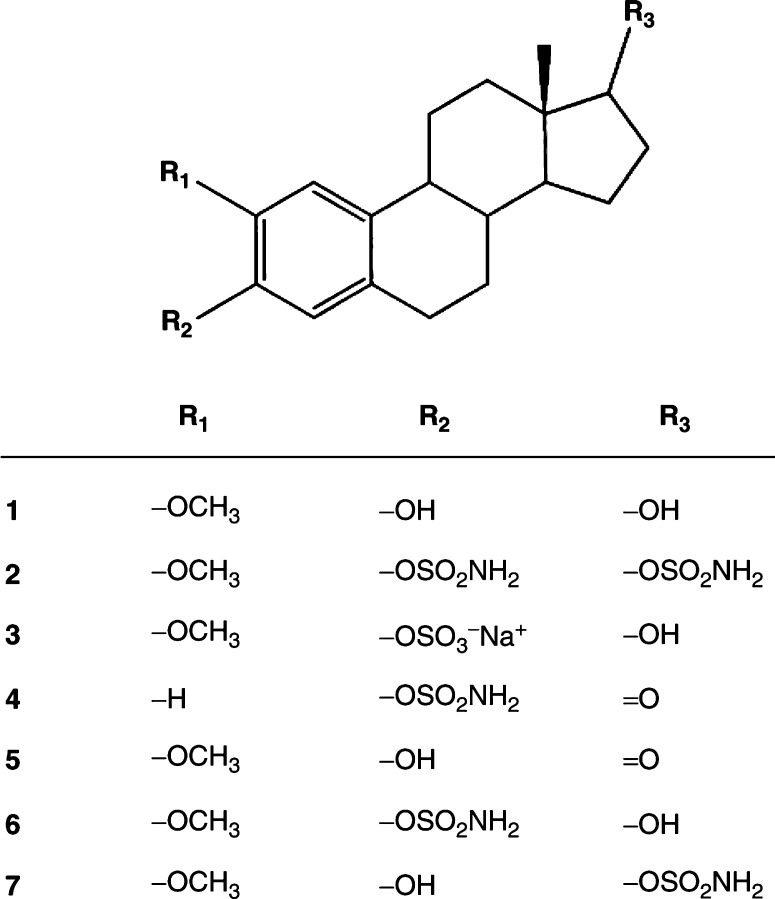
Structures: compound **1**, 2-MeOE2; compound **2**, 2-MeOE2*bis*MATE; compound **3**, 2-MeOE2-3S; compound **4**, EMATE; compound **5**, 2-MeOE1; compound **6**, 2-MeOE2-3MATE; compound **7**, 2-MeOE2-17MATE.

, **1**), a metabolite of oestradiol, is generated *in vivo* by catechol-*O*-methyl transferase, an enzyme which is expressed in a plethora of mammalian tissues including the liver, kidney, brain and red blood cells (Mannisto and Kaakkola, 1999). 2-Methoxyoestradiol has been shown to inhibit the growth of ER+ and ER− breast cancer cell lines and HeLa cells (Seegers *et al*, 1989; Lottering *et al*, 1992; Raobaikady *et al*, 2003). It does not stimulate uterotropic growth, and has a low binding affinity for the oestrogen receptor (Ball and Knuppen, 1980; Merriam *et al*, 1980), and therefore it must act via an oestrogen receptor-independent mechanism (LaVallee *et al*, 2002). It also inhibits the *in vivo* growth of xenografts derived from human MDA-MB-435 melanoma cells, Meth A sarcomas, B16 melanomas and the multiple myeloma cell line KAS-6/1 in immunodeficient mice (Fotsis *et al*, 1994; Klauber *et al*, 1997; Dingli *et al*, 2002). However, comparatively high oral or intraperitoneal doses of 75 and 150 mg kg^−1^ day^−1^, respectively, of 2-MeOE2 were necessary to reduce the growth of melanoma or myeloma tumours (Klauber *et al*, 1997; Dingli *et al*, 2002). The relatively high doses of 2-MeOE2 required to inhibit tumour growth *in vivo* in the mouse may be a corollary of poor gastrointestinal absorption and/or rapid metabolic deactivation of the agent, although this remains to be experimentally determined.

In addition to inhibiting the proliferation of cancer cells, 2-MeOE2 also possesses antiangiogenic properties (Fotsis *et al*, 1994). Inhibition of human umbilical vein endothelial cell (HUVEC) growth is used as a predictive assay of a drug's antiangiogenic potential. The sulphamoylated product of 2-MeOE2, 2-methoxyoestradiol-*bis-*sulphamate (2-MeOE2*bis*MATE, Figure 1, 2), was 60-fold more potent in an *in vitro* HUVEC growth-inhibition assay (Newman *et al*, 2004). 2-Methoxyoestradiol-*bis*-sulphamate has also been shown to inhibit neo-vascularisation *in vivo* in the mouse Matrigel plug model of angiogenesis (Chander *et al*, 2003).

2-MeOE2*bis*MATE is a derivative of oestrone-3-*O*-sulphamate (EMATE, Figure 1, 4) which was originally developed as a steroid sulphatase (STS) inhibitor (Purohit *et al*, 1998). Unexpectedly, EMATE proved to be a potent oestrogen on oral application in rats (Elger *et al*, 1995). The observed oestrogenicity of oestrogen sulphamates, such as EMATE, is thought to be a consequence of their sequestration into red blood cells (RBCs) and slow release of the oestrogen moiety into plasma (Elger *et al*, 1998). It is conceivable that, similarly, the sulphamoylation of 2-MeOE2, and the subsequent uptake of the ester into erythrocytes, may reduce the first pass metabolism of the agent and thus enhance its oral bioavailability. To investigate this postulate we compared the pharmacokinetics of 2-MeOE2*bis*MATE and 2-MeOE2 following administration of a single oral or intravenous dose in rats.

In addition, as part of the pharmacokinetic study, we also examined the metabolism of these two compounds *in vivo*. As sulphamoylated steroids can act as STS inhibitors, the ability of 2-MeOE2*bis*MATE to inhibit the *in vivo* activity of this enzyme was also examined (Reed *et al*, 1996). To compare the abilities of 2-MeOE2 and 2-MeOE2*bis*MATE to inhibit tumour growth *in vivo*, their effects on the growth of xenografts derived from MDA-MB-435 (ER−) human melanoma cells were also examined. This cell line was chosen for *in vivo* studies, as it had previously been used to test the efficacy of 2-MeOE2 (Klauber *et al*, 1997). Since the present study was initiated, it has been revealed that the MDA-MB-435 cell line is not derived from a breast carcinoma, but probably originates from a melanoma (Ellison *et al*, 2002). However, as this cell line is highly metastatic and ER−, it remains a good model for testing agents which should be active against a wide range of hormone-independent cancers.

## MATERIALS AND METHODS

### Chemicals and reagents

The following reagents were purchased from the suppliers listed: high-performance liquid chromatography (HPLC) grade methanol, diethyl ether (Fisher Scientific UK Limited, Loughborough, Leicestershire, UK); Halothane Astra Zeneca, Cheshire, UK); propylene glycol, ammonium sulphate, sodium azide, tetrahydrofuran (THF) (Sigma-Aldrich Comp. Ltd, Poole, Dorset, UK); 17*α*-epitestosterone (Steraloids, Newport, RI, USA); [6,7-^3^H] oestrone sulphate (43-50 Ci mmol^−1^, Perkin-Elmer, Boston, MA, USA). 2-methoxyoestrone (2-MeOE1, Figure 1, 5), 2-MeOE2, 2-MeOE2*bis*MATE, 2-MeOE2-3S (Figure 1, 3), 2-methoxyoestradiol-3-sulphamate (2-MeOE2-3MATE, Figure 1, **6**) and 2-methoxyoestradiol-17-sulphamate (2-MeOE2-17MATE, Figure 1, 7) were synthesised from oestrone. All new compounds exhibited spectroscopic and analytical data in accordance with their structure. Full details of their synthesis will be reported elsewhere.

### *In vivo* pharmacokinetic and metabolism studies

Female *Wistar* (155–165 g) rats were purchased from Charles River UK Ltd (Margate, Kent, UK) and housed in a dedicated animal facility. Rats received RM1 rodent maintenance diet (SDS, Kent, UK), water *ad libitum*, and were maintained in positive pressure isolators under a 12 h light–dark cycle. These experiments were carried out under conditions that complied with institutional requirements. Rats received 2-MeOE2 or 2-MeOE2*bis*MATE (10 mg kg^−1^, oral or intravenous), with control animals receiving vehicle only (propylene glycol: THF, 9 : 1 v v^−1^). There were two reasons for selecting 10 mg kg^−1^ for both oral and intravenous dosing. Firstly, a single dose of EMATE administered by these routes has been shown to inhibit rat liver sulphatase by at least 99% (Purohit *et al*, 1995). Secondly, administration of this dose was found to elicit sufficiently high levels of the agents for their detection in plasma. Rats were subjected to terminal anaesthesia (Halothane) and blood removed by cardiac puncture at 5, 15 and 30 min and 1, 3, 8 and 24 h after intravenous administration, and 15 and 30 min and 1, 3, 8, 24 and 48h following oral administration of 2-MeOE2 or 2-MeOE2*bis*MATE. Plasma was prepared from whole blood by centrifugation (2800 **g**, 4°C, 15 min). Plasma (0.5 ml) was extracted with diethyl ether (4 ml) and frozen in a methanol : solid carbon dioxide mixture. 17*α*-Epitestosterone (28 *μ*g ml^−1^) was used as an internal standard after purification by HPLC. The organic phase was decanted to a fresh tube and evaporated to dryness under a stream of air at room temperature. The extraction efficiencies for 2-MeOE2 and 2-MeOE2*bis*MATE from plasma were 71±5 and 75±4% (*n*=6), respectively. The residues were stored at −20°C until analysis by HPLC.

### HPLC analysis

2-MeOE2*bis*MATE was separated from its putative metabolites, 2-MeOE2, 2-MeOE2-3MATE and 2-MeOE2-17MATE, using a modified version of a reversed-phase HPLC method described previously (Hildago Aragones *et al*, 1996). An Agilent 1100 (Cheshire, UK) autosampler, photodiode array detector and solvent delivery system were used. The agents were separated from endogenous plasma components by an isocratic mobile phase consisting of 58% methanol in 0.02 M ammonium sulphate. Sodium azide (1 mM) was added to the mobile phase in order to decrease microbial growth. Extracted samples were reconstituted in mobile phase and aliquots of 100 *μ*l were injected on to a C3-phenyl column (250 × 5 mm, 5 *μ*m) purchased from Phenomenex (Cheshire, UK). 2-Methoxyoestradiol-*bis*-sulphamate, 2-MeOE2 and their metabolites were analysed with a photodiode array detector with detection at 285 nm. The method was validated by spiking plasma with 2-MeOE2*bis*MATE (960 ng ml^−1^). The inter-day and intra-day coefficients of variation were 8.2% (*n*=6) and 3.8% (*n*=6), respectively. Quantification was achieved by spiking plasma with 2-MeOE2*bis*MATE and the internal standard 17*α*-epitestosterone, extraction with diethyl ether and subsequent analysis by HPLC. Plasma calibration curves were found to be linear from 40 to 9000 ng ml^−1^. The limits of detection (LODs) and quantification of 2-MeOE2*bis*MATE in plasma were 11 and 40 ng ml^−1^, respectively.

### Pharmacokinetic analysis

Pharmacokinetic parameters were calculated using WinNonlin software (Pharsight Corporation, Mountview, CA, USA). The area under the curve (AUC) was calculated using the linear trapezoidal method, with extrapolation of the terminal phase to infinity. Other parameters calculated were: distribution and elimination rate constants (*α* and *β*); total body clearance (*Cl*)=dose/AUC; volume of distribution (Vd)=*Cl*/*β;* distribution half-life (*t*_1/2_*α*)=0.693/*α*; elimination half-life (*t*_1/2_*β*)=0.693/*β*; bioavailability (% *F=*(AUC_oral_/AUC_*i.v*_) × 100).

### Liver oestrone sulphatase activity

Livers were obtained from animals administered with 2-MeOE2 or 2-MeOE2*bis*MATE either orally or intravenously, to assess their effects on steroid sulphatase activity. Steroid sulphatase activity was measured as described previously (Purohit *et al*, 1995).

### *In vivo* inhibition of tumour growth

To compare the anticancer effects of 2-MeOE2 and 2-MeOE2*bis*MATE, xenografts derived from MDA-MB-435 (ER−) human melanoma cells were transplanted into female nude mice with eight mice per group. These studies were carried out by Anti-Cancer Inc. (San Diego, CA, USA). Treatment was initiated when tumour volumes reached 100–200 mm^3^. Drugs were dissolved in a minimum volume of THF, diluted with propylene glycol and administered at 20 mg kg^−1^, oral, daily for 28 days. The length (*l*) and width (*w*) of tumours was measured at weekly intervals, from which the tumour volumes were calculated using the formula (*l × w*^*2*^*/2*). Monitoring of tumour volumes continued for a further 28-day period after the end of drug administration. The body masses of control and treated animals were also measured at weekly intervals as an indicator of any toxicity that might be associated with the use of these drugs.

### Statistics

Student's *t*-test was used to assess the significance of differences in tumour volumes between control and treated animals.

## RESULTS

### HPLC analysis

A reproducible, robust and sensitive analytical method was developed for the detection of 2-MeOE2 and 2-MeOE2*bis*MATE in plasma. The method facilitated separation of these agents from the putative metabolites 2-MeOE1, 2-MeOE2-17MATE and 2-MeOE2-3MATE, the internal standard, 17*α*-epitestosterone, and endogenous plasma components.

### Pharmacokinetics of 2-MeOE2 and 2-MeOE2*bis*MATE

**Figure 2:**
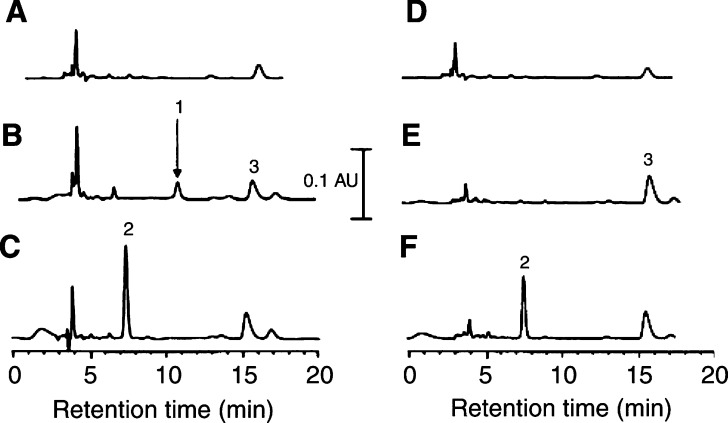
HPLCs of diethyl ether plasma extracts. Plasma was prepared from whole blood following administration of intravenous (**A**) propylene glycol, (**B**) 2-MeOE2, (**C**) 2-MeOE2*bis*MATE or oral, (**D**) propylene glycol, (**E**) 2-MeOE2 and (**F**) 2-MeOE2*bis*MATE. The blood was removed from animals 0.25 and 3 h after intravenous and p.o. dosing, respectively. The identities of 2-MeOE2, 2-MeOE2*bis*MATE and 17*α*-epitestosterone (internal standard) as being peaks 1, 2 and 3, respectively, were corroborated by co-elution with authentic standards and liquid chromatography-mass spectrometry. For details of extraction and HPLC analysis, see ‘Materials and Methods’. The peak eluting at 3.9 min was an endogenous plasma component.

**Figure 3:**
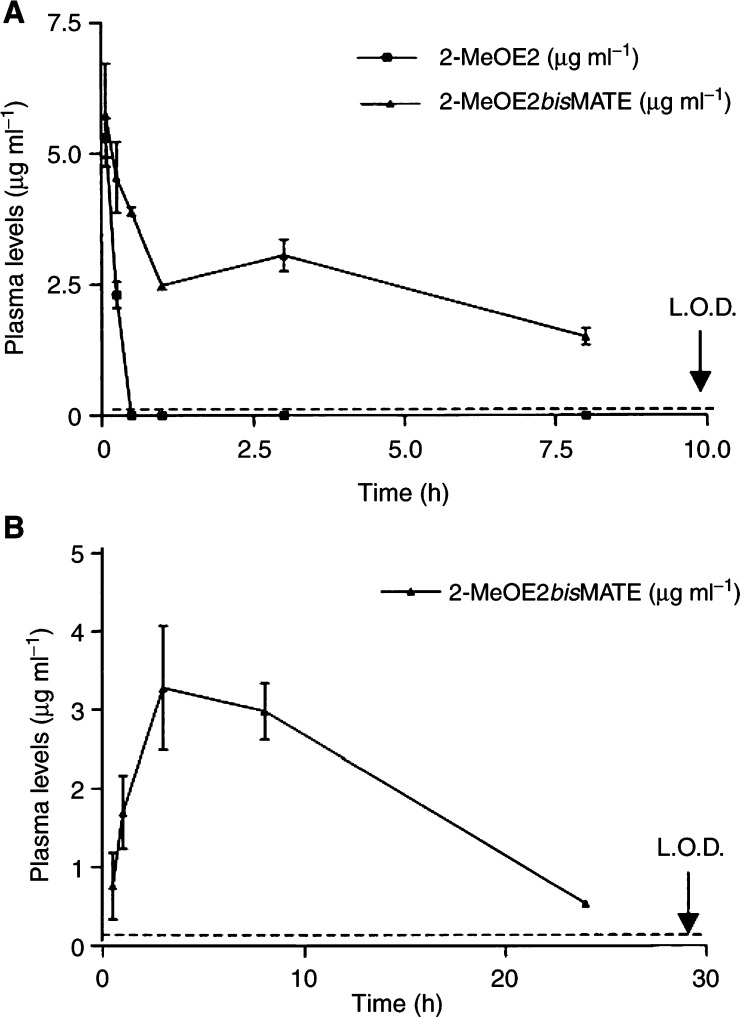
Concentrations of 2-MeOE2 and 2-MeOE2*bis*MATE in rat plasma following a single intravenous (**A**) or oral (**B**) bolus of drug (10 mg kg^−1^) in THF/propylene glycol. The values shown are the means ±s.e.m. (*n*=3). 2-Methoxyoestradiol was below the LOD (11 ng ml^−1^) in plasma after oral administration. Where no error bars are shown, the coefficient of variation is <10%.

In order to compare the pharmacokinetics of 2-MeOE2 and 2-MeOE2*bis*MATE, rats were administered with either a single intravenous or oral dose of the agents. Using the HPLC analytical method developed, it was possible to detect both compounds in plasma after intravenous administration (Figure 2B, C

), but only 2-MeOE2*bis*MATE was detected after oral administration (Figure 2E, F). The identities of these agents were corroborated by electrospray ionisation liquid chromatography-mass spectrometry in the selected ion mode (results not shown). When 2-MeOE2 was given to rats as an intravenous bolus, the agent was found to be rapidly removed from the plasma (Figure 3A

) and was below the LOD (11 ng ml^−1^) 1 h after administration of the dose. Consequently, it was not possible to determine the pharmacokinetic parameters for 2-MeOE2. In contrast, when 2-MeOE2*bis*MATE was administered as an intravenous bolus, the parent compound was quantifiable in plasma 8 h after administration of the agent (1.52±0.10 *μ*g ml^−1^), although it was not detectable by 24 h. The decrease in plasma concentration of 2-MeOE2*bis*MATE followed a biexponential pattern with an initial distribution half-life (t_1/2_*α*) of 0.22±0.10 h and a terminal half-life (*t*_1/2_*β*) of 8.50±1.16 h. The area under the plasma concentration–time curve (AUC) was 513.99±153.88 h *μ*g ml^−1^. Pharmacokinetic parameters after oral administration were calculated using an extravascular noncompartmental model. Maximum levels of 2-MeOE2*bis*MATE in plasma (*C*_max_=3.90±0.25 *μ*g ml^−1^) were reached 3 h after oral administration of the agent (Figure 3B and Table 1

**Table 1:** Summary of 2-MeOE2*bis*MATE pharmacokinetic data after bolus intravenous or oral administration

**PK parameters**	**Intravenous**	**Oral**
Cmax (*μ*g ml^−1^)	6.55±0.92	3.90±0.25
*t*_1/2_*α* (h)	0.22±0.10	—
*t*_1/2_*β* (h)	8.50±1.16	6.51±1.09
AUC (h *μ*g ml ^−1^)	513.99±153.88	388.84±28.84
Vd (l)	0.28±0.09	0.35±0.02
Cl (l h^−1^)	0.05±0.01	0.036±0.001
% F (bioavailability)	—	85.19±16.09

Values shown are mean±s.e.m. (*n*=3).

) with 0.53±0.04 *μ*g ml^−1^ still detectable after 24 h. A terminal plasma half-life of 6.51±1.09 h with an AUC of 388.84±28.84 h *μ*g ml^−1^ was achieved. The bioavailability for 2-MeOE2*bis*MATE based on AUC after intravenous or oral administration was 85.19±16.09%. The putative metabolites of 2-MeOE2*bis*MATE, 2-MeOE2-17MATE, 2-MeOE2-3MATE and 2-MeOE2 were not above the LOD in plasma.

### Inhibition of liver sulphatase

Hepatic sulphatase activity was inhibited by 99.5±0.5% compared with the activity measured in control animals, following administration of a single oral or intravenous dose of 2-MeOE2*bis*MATE. This level of inhibition of sulphatase activity was maintained for 48 h following administration of 2-MeOE2*bis*MATE. 2-Methoxyoestradiol did not affect rat liver sulphatase activity (data not shown).

### *In vivo* inhibition of tumour growth

**Figure 4:**
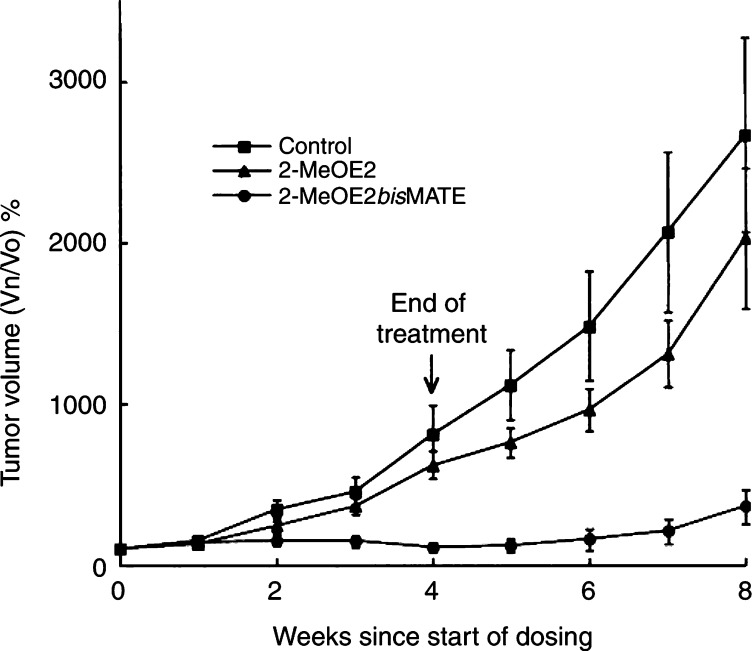
*In vivo* effect of 2-MeOE2 or 2-MeOE2*bis*MATE on the growth of MDA-MB-435 (ER−) xenografts in nude mice. Vehicle (THF/propylene glycol) or compounds were administered daily (20 mg kg^−1^, oral) for 28 days. Tumour volumes were monitored at weekly intervals for the duration of drug administration and for a further 4-week period after the end of drug administration (means±s.e.m., *n*=8). Results are expressed as percentage change in tumour volumes detected at weekly intervals. At this dose, 2-MeOE2 had no significant effect on tumour growth during the treatment or post-dosing periods. For 2-MeOE2*bis*MATE-treated animals, tumour volumes were significantly smaller than those in control animals by week 2 (*P*<0.05) and remained significantly smaller (*P*<0.01) for the rest of the study period.

**Figure 5:**
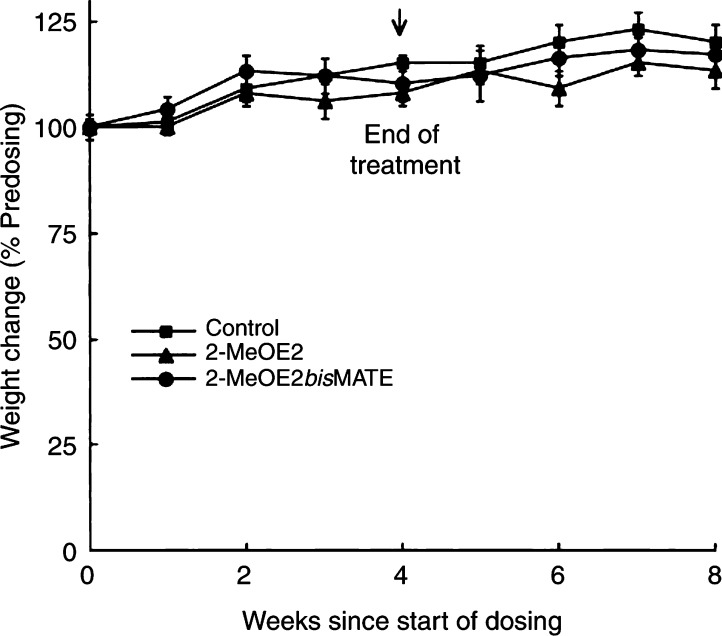
Changes in weights of control animals and those administered 2-MeOE2 or 2-MeOE2*bis*MATE. Animals were weighed at weekly intervals and the results are expressed as the percentage change compared with their pre-dosing weights (means±s.e.m., *n*=8).

To compare the effects of 2-MeOE2 and 2-MeOE2*bis*MATE on *in vivo* tumour growth in nude mice that had been inoculated with xenografts derived from MDA-MB-435 human melanoma cells, animals were treated with drugs (20 mg kg^−1^, oral) daily for 28 days (Figure 4

). At this dose, 2-MeOE2 had no significant effect on tumour growth during the treatment or post-dosing periods. In contrast, by week 2 of dosing, the tumour volumes of mice receiving 2-MeOE2*bis*MATE were significantly smaller (*P*<0.05) than those of the control group. Tumour volumes for mice treated with 2-MeOE2*bis*MATE then remained significantly smaller (*P*<0.01) for the remaining period of the study. At 4 weeks, at the end of the dosing period, the mean tumour volumes in mice receiving 2-MeOE2*bis*MATE were 14% of that of tumour volume in the control animals. This difference (86%) in tumour volumes between the two groups was maintained for a further 4 weeks after the cessation of drug administration. No significant differences in the change in weights between the control and treated animals were detected during the treatment or post-dosing periods (Figure 5

).

## DISCUSSION

The sulphamoylated oestrogen derivative 2-MeOE2*bis*MATE has previously been shown to be a more potent inhibitor of cancer cell proliferation than its parent compound, 2-MeOE2 (Raobaikady *et al*, 2003). Furthermore, in *in vitro* models of angiogenesis, 2-MeOE2*bis*MATE proved to be considerably more potent as an inhibitor of angiogenesis than 2-MeOE2 (Newman *et al*, 2004). The main finding to emerge from the present *in vivo* study goes some way to offer an explanation for the enhanced potency of 2-MeOE2*bis*MATE. In this study, the plasma concentrations of both drugs were determined after oral or intravenous dosing, together with an examination of the duration for which the compounds and putative metabolites were detectable in plasma. After intravenous administration of 2-MeOE2, the compound was rapidly cleared from the plasma, suggesting metabolic removal. 2-Methoxyoestradiol eluded detection in plasma after its oral administration and, by 1 h after intravenous dosing, levels were below the LOD for the assay. In contrast, significant concentrations of 2-MeOE2*bis*MATE were detectable in plasma after its oral or intravenous administration with 450 ng ml^−1^ still being detectable at 24 h after oral dosing. Recently, a daily oral dose of 1000 mg 2-MeOE2 was given to 24 patients with advanced metastatic breast cancer (Sledge *et al*, 2002; Lakhani *et al*, 2003). It was shown that 2-MeOE2 was metabolised to 2-MeOE1 and 2-MeOE2/2-MeOE1 glucuronide and sulphate conjugates. Demethylated 2-MeOE1 (2-OHE1) has also been measured in the urine of humans administered with [^3^H]2-MeOE2, although this metabolite could not be detected in plasma (Longcope *et al*, 1980).

After intravenous administration of 2-MeOE2, its half-life in plasma was approximately 14 min. Pharmacokinetic studies in rats indicated that 2-MeOE2*bis*MATE was detectable in plasma for up to at least 24 h after oral administration. The calculated bioavailability for 2MeOE2*bis*MATE from the AUC was 85.2%. A possible explanation for this finding may be that, initially, there is sequestration of 2-MeOE2*bis*MATE into blood components, possibly RBCs (unpublished data), or binding to plasma proteins, resulting in high AUC orally. This may, in part, account for the slow clearance rates and the high volume of distribution achieved by both intravenous and oral administration. Nevertheless, it is apparent that 2-MeOE2*bis*MATE has a higher level of bioavailability after oral administration, compared with that of the non-sulphamoylated 2-MeOE2.

Further evidence of the enhanced potency of 2-MeOE2*bis*MATE compared with that of 2-MeOE2 was obtained from the *in vivo* study carried out in nude mice bearing xenografts of MDA-MB-435 tumours. 2-Methoxyoestradiol had little effect on the growth of these tumours. In contrast, for animals receiving 2-MeOE2*bis*MATE, tumour growth was almost completely inhibited for the 4-week treatment period, and its growth-inhibiting effects were maintained for 4 weeks after the cessation of dosing. It is evident from the present study that 2-MeOE2*bis*MATE can inhibit *in vivo* tumour growth. Further studies will be required to ascertain whether it is acting by inhibition of angiogenesis or induction of apoptosis or, as most likely, a combination of both mechanisms. No decreases in body weights were detected in animals receiving either 2-MeOE2 or 2-MeOE2*bis*MATE, indicating that, at the dose tested, these compounds have little or no toxicity.

From these studies, it is evident that 2-MeOE2*bis*MATE is resistant to metabolism *in vivo*, and this finding goes some way to account for its enhanced potency compared with 2-MeOE2*. In vivo*, the C3 phenolic group and 17*β* hydroxyl group of 2-MeOE2 are likely to be subjected to rapid conjugation or oxidation, thus reducing the potency of this compound as an anticancer and antiangiogenic drug. Several derivatives of 2-MeOE2 have been developed, such as 2-methoxymethyloestradiol and a series of 2-methoxytetrane oestradiols, which are more potent inhibitors of *in vitro* cancer cell proliferation than 2-MeOE2 itself (Bruggemeier *et al*, 2001; Tinley *et al*, 2003). However, as these compounds retain the C3/17*β* configuration of 2-MeOE2, it is unlikely that they will prove to be more potent *in vivo* and will undergo the same route of rapid inactivation.

EMATE was previously found to have a half-life of 4.5 h in plasma (Hildago Aragones *et al*, 1996), and this compares with the much shorter half-life for unconjugated oestrogen of 20–30 min (Ruder *et al*, 1972). Like EMATE, 2-MeOE2*bis*MATE is a potent steroid sulphatase inhibitor and, in the present study, liver sulphatase was rapidly inactivated after its oral or intravenous administration. Almost complete inhibition of steroid sulphatase activity (>99%) was achieved within 5 and 15 min of intravenous and oral dosing, respectively. Inhibition of steroid sulphatase activity will prevent removal of the sulphamoyl group at the C3 position, thus greatly prolonging the plasma, and presumably tissue, concentrations of the active sulphamoylated drug. The relatively high plasma concentrations of 2-MeOE2*bis*MATE (up to 5 *μ*M) detected are within the range that has been shown to inhibit cancer cell and HUVEC proliferation *in vitro* (Newman *et al*, 2004). Thus, sulphamoylation of 2-MeOE2 yields a drug that is resistant to *in vivo* metabolism. *In vivo,* its ability to inhibit STS appears to block its de-sulphamoylation, ensuring that the biologically active drug is available to exert its effects for a prolonged period of time. The findings from *in vitro* studies that 2-MeOE2*bis*MATE is more potent than 2-MeOE2, together with the results of the *in vivo* studies showing no evidence of conversion to 2-MeOE2, indicate that it is not acting as a pro-drug for the formation of 2-MeOE2.

There is currently considerable interest in exploring the mechanisms by which 2-MeOE2 and its sulphamoylated derivates act to inhibit cancer cell growth and angiogenesis. 2-Methoxyoestradiol is an endogenous oestrogen metabolite, and it has been suggested that it may be the body's natural anticancer metabolite (Zhu and Conney, 1998). This contention is supported by a significant body of research carried out by Bradlow *et al* (1998), who have convincingly demonstrated that production of 2-MeOE2 is increased in women at low risk of breast cancer. 2-Methoxyoestradiol and its sulphamoylated derivatives are thought to bind to the colchicine-binding site of tubulin, where they act to alter microtubule dynamics, leading to activation of the intrinsic apoptotic pathway via BCL-2 phosphorylation (D'Amato *et al*, 1994; MacCarthy-Morrogh *et al*, 2000). In addition, it is possible that, like 2-MeOE2, the sulphamoylated derivatives will also act to induce apoptosis via activation of the extrinsic apoptotic pathway, which involves upregulation of death receptors, such as DR5 (LaVallee *et al*, 2003).

*In vitro*, 2-MeOE2 has been shown to inhibit the proliferation of a wide range of cancer cells including those derived from the breast, prostate, ovary, pancreas, lung and multiple myelomas. At relatively high doses (75–150 mg kg^−1^), its efficacy in mouse models against melanomas, Meth A sarcomas and multiple myelomas has been demonstrated (Klauber *et al*, 1997; Dingli *et al*, 2002). As shown in the present *in vivo* efficacy study at a dose of 20 mg kg^−1^, while 2-MeOE2 was ineffective, 2-MeOE2*bis*MATE almost completely blocked tumour growth. The identification of 2-MeOE2*bis*MATE as a potent anticancer, antiangiogenic drug that is orally available, yet resistant to metabolism, should make it a good candidate for development for cancer therapy.
